# Semantic congruency modulates the speed-up of multisensory responses

**DOI:** 10.1038/s41598-023-50674-4

**Published:** 2024-01-04

**Authors:** Kalvin Roberts, Ines Jentzsch, Thomas U. Otto

**Affiliations:** School of Psychology and Neuroscience, University of St. Andrews, St. Mary’s Quad, South Street, St. Andrews, KY16 9JP UK

**Keywords:** Sensory processing, Human behaviour, Perception, Decision, Computational neuroscience

## Abstract

Responses to multisensory signals are often faster compared to their unisensory components. This speed-up is typically attributed to target redundancy in that a correct response can be triggered by one or the other signal. In addition, semantic congruency of signals can also modulate multisensory responses; however, the contribution of semantic content is difficult to isolate as its manipulation commonly changes signal redundancy as well. To disentangle the effects of redundancy and semantic congruency, we manipulated semantic content but kept redundancy constant. We presented semantically congruent/incongruent animal pictures and sounds and asked participants to respond with the same response to two target animals (cats and dogs). We find that the speed-up of multisensory responses is larger for congruent (e.g., barking dogs) than incongruent combinations (e.g., barking cats). We then used a computational modelling approach to analyse audio-visual processing interferences that may underlie the effect. Our data is best described by a model that explains the semantic congruency modulation with a parameter that was previously linked to trial sequence effects, which in our experiment occur from the repetition/switching of both sensory modality and animal category. Yet, a systematic analysis of such trial sequence effects shows that the reported congruency effect is an independent phenomenon. Consequently, we discuss potential contributors to the semantic modulation of multisensory responses.

## Introduction

Multisensory stimuli benefit perception compared to their unisensory constituents. For example, responses to multisensory stimuli (e.g., seeing and hearing an animal simultaneously) are faster than responses to unisensory constituents alone (either seeing or hearing the animal), which is typically referred to as the *redundant signal effect* (RSE^1–5^). Although reliably replicated, the mechanisms behind these benefits remain a central topic in multisensory research.

One key question is which combination of stimuli leads to multisensory benefits. The unity or common origin assumption—the assumption that two or more sensory cues ‘belong’ together—has been used over decades to investigate the relationship between stimuli and multisensory benefits^6^. A close spatiotemporal coincidence of stimuli is one component of establishing a common origin^7–9^. In addition, higher-order cognitive factors such as semantic congruency^10–13^ and crossmodal correspondences have been investigated^14,15^. When looking at semantic congruency, stimuli represent either the same or different objects (e.g., an image of a dog paired either with a dog’s “woof” or a cat’s “meow” sound). With respect to multisensory benefits, it was reported that faster multisensory responses are only present when stimuli are semantically congruent^10,12,13,16^. However, benefits can also occur in multisensory conditions with semantically incongruent stimuli^17–19^, making the contribution of semantic congruency unclear.

To test the effect of semantic congruency on multisensory responses, for example, Laurienti et al.^10^ asked participants to respond to a target colour (e.g., red), which was presented either in vision (as a coloured circle) and/or in audition (as a vocalisation of the colour name). When presented together, stimuli could either be semantically congruent (e.g., a red circle with the vocalisation “red”) or incongruent in that a target colour stimulus was paired with a non-target stimulus (e.g., a red circle with the vocalisation “green”). A speed-up of responses to multisensory stimuli is only observed with semantically congruent stimuli, whilst responses to incongruent conditions are even slower than those of the unisensory constituents. Likewise, using animal images and their vocalisations, Molholm et al.^12^ used an analogous paradigm to investigate multisensory object recognition with stimulus combinations that were either semantically congruent (e.g., a line image of a dog and a “woof” sound) or incongruent (e.g., a line image of a dog and a “meow” sound). Again, while responses to congruent stimuli showed a speed-up, responses in incongruent conditions did not, an effect replicated in similar studies^13,16^. These findings may suggest that only congruent stimuli facilitated faster multisensory responses.

Common in these studies is that semantically congruent conditions present redundant targets, whereas incongruent conditions do not. As participants are asked to respond to a specific stimulus feature in one or the other modality, only one target is present in semantically incongruent conditions, but two targets are present in semantically congruent conditions. Targets are here redundant in the sense that detecting one or the other signal is sufficient for a correct response. For example, when participants are asked to respond to “dogs”, the semantically congruent combination of a dog image with a barking sound presents two target signals, one in audition and one in vision. In contrast, the semantically incongruent combination of a dog image with a meowing sound presents only a visual target signal. Hence, in addition to the semantic congruency of stimuli, the difference in target redundancy may be a critical factor that modulates responses to multisensory signals in the above studies.

The presentation of redundant signals is expected to be beneficial because a multisensory response can be evoked by the faster of two parallel unisensory decision processes (e.g., one for audition and one for vision, each checking if a target is present, yes or no?). This simple idea is formalised in so-called race models, which assume that parallel unisensory decision units are coupled by a logic OR gate^20^. The model predicts a benefit that can be quantified by using probability summation as a combination rule. As an illustration, when playing dice, it is more likely to obtain a small number when throwing two dice and picking the smaller number compared to rolling only one. For example, when rolling one die, the probability of getting a “1” is 1/6, or 16.7%. When rolling two dice, the probability of obtaining at least one “1” is 1/6 + 1/6 − 1/36, or 30.6%. Likewise, the probability of getting smaller (faster) response times is higher when two redundant targets are presented, compared to only one target (as was the case with incongruent conditions in the above-mentioned studies). Hence, it is critical to consider predicted benefits due to target redundancy when studying the effect of semantic congruency on multisensory responses.

There are two ways to control the effect of target redundancy in experiments that investigate the effect of semantic congruency on multisensory responses. First, to remove redundancy from both congruent and incongruent conditions, participants can be asked to respond only to one modality (e.g., to a dog image, but not to a “woof” sound). Congruent multisensory conditions are non-redundant as the secondary stimulus is task irrelevant. In such experiments, incongruent response times do not differ from those in unisensory conditions, whilst responses to semantically congruent signals are considerably faster^21,22^. Second, to present redundant targets in both congruent and incongruent conditions, participants can be asked to respond to semantically different target stimuli with the same motor response (e.g., respond to any dog *or* cat, where a meowing dog presents targets both in vision and audition). Using this design, and in line with typical RSE experiments, responses to congruent and incongruent multisensory signals are often faster than the unisensory components^11,17–19^. Interestingly, the magnitude of this RSE is still modulated by the semantic congruency of signals, however there is conflicting evidence whether the RSE is larger in congruent^17^ or incongruent conditions^19^. Moreover, a systematic investigation of how semantic congruency modulates the multisensory responses beyond the RSE as predicted by probability summation is lacking. Hence, the effect of semantic congruency, irrespective of differences in redundancy, remains elusive.

**Figure 1:**
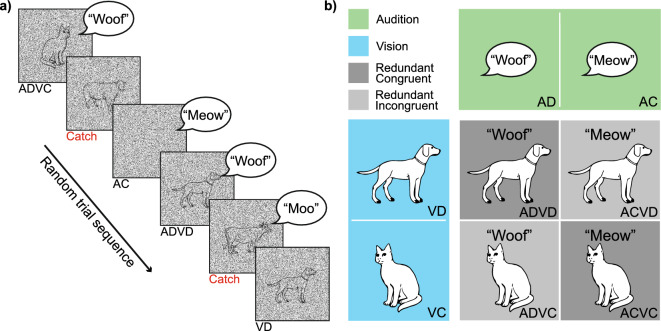
Experimental design. (**a**) Example trial sequence of the redundant signal paradigm. Participants are asked to detect any cat (C) or dog (D). Target trials were either auditory (A), visual (V), or redundant (AV). Critically, redundant audio-visual targets could be either semantically congruent (e.g., a dog image with a “woof” sound, ADVD) or incongruent (e.g., a cat image with a “woof” sound, ADVC). Responses were not required for catch trials showing other animals (red). (**b**) 2 × 2 design of target conditions. Each square presents one of eight target conditions, which were randomly presented. Redundant conditions use all combinations of unisensory cat/dog stimuli, resulting in two semantically congruent and two incongruent combinations. Images adapted from Snodgrass and Vanderwart^25^.

To systematically study the effect of semantic congruency, we here test the RSE with animal images and vocalisations as used previously^11,12,16^. Following the second design described above, we asked participants to respond with the same motor response to any dog or cat stimulus (whether presented in vision, audition, or simultaneously in both modalities; Fig. 1a). Participants were asked to withhold a response to any other animal (i.e., in trials presenting cows, sheep, birds, frogs, monkeys or chickens). Hence, there were in total four unisensory target conditions (cat and dog images, “meow” and “woof” sounds). Using a 2 × 2 design with visual and auditory animals as factors, we generated four multisensory conditions that always present redundant targets (Fig. 1b). Two conditions were semantically congruent (barking dog, meowing cat), and two were semantically incongruent (meowing dog, barking cat). This design allows us to study the effect of semantic congruency on the RSE relative to predictions that account for target redundancy using probability summation^20^. Following this initial analysis, we then investigate trial sequence effects as potential factors underlying any such modulation using a model-based approach^23,24^.

## Results

### Semantic congruency modulates the redundant signal effect

**Figure 2:**
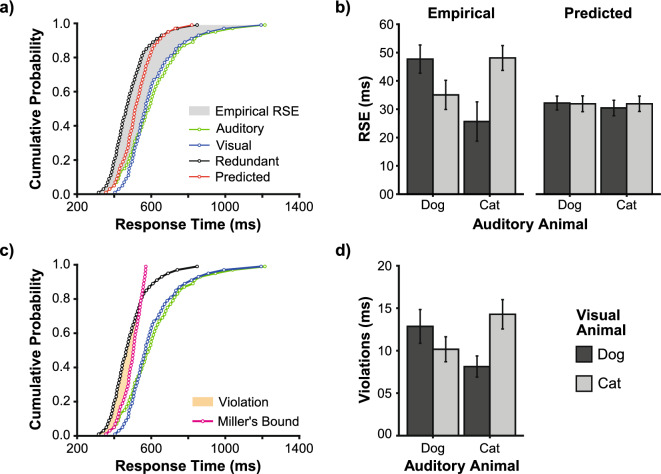
Congruency effects (**a**) Measuring the RSE. Example data from one participant showing cumulative response time distributions in unisensory (auditory, visual) and redundant conditions (audio-visual). The RSE can be quantified as the area between the faster unisensory and the redundant distribution (grey area). In addition, a parameter-free prediction can be computed using probability summation (red). The predicted RSE can then be quantified analogously to the empirical RSE. (**b**) Empirical and predicted RSE for each combination of auditory and visual animal type (cat, dog). The semantic congruency of animal stimuli modulated the empirical RSE; no such effect was observed in the prediction RSE. (**c**) Testing race models. Miller’s bound (pink) is computed by the sum of cumulative response time distributions in the two unisensory conditions (auditory and visual). It is violated if the empirical distribution with redundant signals (black) crosses towards faster response times, which can be quantified by the area between the two (yellow). Example data as shown in panel (**a**). (**d**) Violations of Miller’s bound. Both semantically congruent and incongruent conditions violated the bound. The magnitude of violations was modulated by semantic congruency. Mean (± SEM) of 21 participants.

To quantify the RSE, we analysed cumulative response time distributions (Fig. 2a). As a typical finding, responses to redundant signals are on average faster than responses to the unisensory component signals, where their distribution is located to the left of the unisensory distributions (first-order stochastic dominance). The empirical RSE can be calculated by the area between the distribution with redundant signals and the fastest responses from the two unisensory distributions (shaded area in Fig. 2a). As an advantage of this analysis, unisensory response time distributions and probability summation allow computing a parameter-free model prediction of the distribution with redundant signals (red distribution in Fig. 2a). Analogous to the empirical RSE, this model prediction allows calculating the predicted RSE as expected from target redundancy. The prediction can reveal changes in the RSE that may arise due to performance differences across unisensory conditions (e.g., differences between dog and cat processing).

Using this measure of the RSE, we performed a 2 × 2 × 2 repeated measures ANOVA with the factors data type (empirical, predicted), visual animal (dog, cat) and auditory animal (dog, cat; Fig. 2b). We found a significant three-way interaction (F_(1,20)_ = 35.21, *p* < 0.001, η_p_^2^ = 0.64), which can be explained by a significant two-way interaction between auditory and visual animals in the empirical RSE (F_(1,20)_ = 31.63, *p* < 0.001, η_p_^2^ = 0.61), which is not present in the predicted RSE (F_(1,20)_ = 1.86, *p* = 0.187, η_p_^2^ = 0.09). The significant two-way interaction in the empirical data revealed a semantic congruency effect where the RSE was largest when auditory stimuli are combined with congruent (47.90 ± 4.56 ms; mean ± SEM) compared to incongruent visual stimuli (30.36 ± 5.55 ms; see Fig. 2a). The RSEs in both congruent conditions (ADVD and ACVC) were larger than in both incongruent conditions (ADVC and ACVD; all *p* ≤ 0.002). In contrast, the predicted RSE shows no differences between the semantically congruent (32.06 ± 2.45 ms) and incongruent condition (31.18 ± 2.61 ms). There was a main effect of data type (F_(1,20)_ = 4.49, *p* = 0.047, η_p_^2^ = 0.18); however, this main effect was driven by the three-way interaction effect as incongruent conditions showed no difference between empirical and predicted RSE (all *p* ≥ 0.407). No further main effects or interactions were significant (all *p* ≥ 0.084). These results indicate that the RSE is influenced by the semantic congruency between auditory and visual animal stimuli, with a more substantial effect observed for congruent pairings. This pattern, which is not present in the predicted RSE, highlights the role of congruency in modulating the RSE.

Miller^3^ developed an analysis providing an upper bound for the response time of redundant signals, consistent with a race model where the faster of two parallel sensory decisions prompts a response. This “Miller’s bound” is given by the sum of individual sensory response probabilities, represented by the pink distribution in Fig. 2c^26^. A violation of this bound, shown by faster redundant response times and indicated by the shaded area in Fig. 2c, shows that the presence of a signal in one modality influences the processing of a signal in the other modality within the race architecture, or that the race architecture is violated altogether, or both^3,27–32^. We tested whether the semantic congruency effect would also be observed in violations of Miller’s bound (Fig. 2d). We conducted a 2 × 2 repeated-measures ANOVA with auditory (dog, cat) and visual animal (dog, cat) as factors. We found a significant intercept (F_(1,20)_ = 76.92, *p* < 0.001, η_p_^2^ = 0.79) indicating that Miller’s bound is generally violated (grand mean, 11.36 ± 1.30 ms), which is further corroborated in that responses in all four conditions (both congruent and incongruent) violated Miller’s bound (all *p* < 0.001). Further, we found a two-way interaction effect (F_(1,20)_ = 17.82, *p* < 0.001, η_p_^2^ = 0.47), showing that violations of Miller’s bound are larger in semantically congruent (13.57 ± 1.59 ms) than in incongruent conditions (9.15 ± 1.17 ms). No other effects reached significance (all* p* ≥ 0.210).

Together, both the overall RSE and violations of Miller’s bound were modulated by semantic congruency. Critically, race models cannot accommodate this congruency effect without assuming processing interactions, which we try to identify in the following.

### Effects of trial sequence on response times

Trial sequence effects have been proposed to mediate larger than predicted RSEs^23,33^ and it is known that response times in the redundant signal paradigm are typically affected by modality switching^3,23,33–37^. Here, we investigate whether trial sequence effects modulate the semantic congruency effect found in response times. Modality switching alone cannot explain the congruency modulation of the RSE, as both congruent and incongruent trials are equally affected by modality switching, where at least one modality is repeated. However, an additional effect of animal switching is present that has the potential to modulate congruency effects. Consequently, we conducted three trial sequence analyses, taking into account the response time of the current trial as a function of the signal(s) presented in the preceding trial.

**Figure 3:**
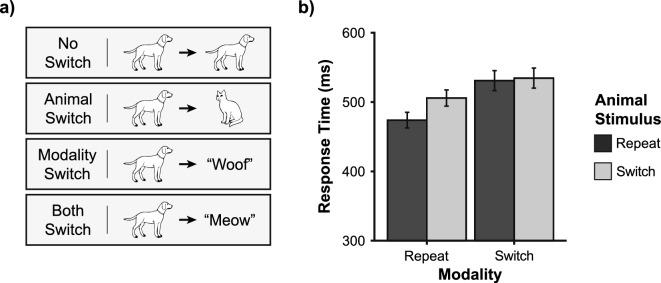
Sequential effects in unisensory response times. (**a**) Basic switch conditions. No switch occurs when a signal from the previous trial is repeated. An animal switch occurs when the animal category is changed but not the sensory modality. A modality switch occurs when the sensory modality is changed but not the animal category. A complete switch occurs when both animal category and sensory modality change. Only four examples of unisensory trial sequences are shown. (**b**) Unisensory response time as a function of modality and animal switch. Mean (± SEM) of 21 participants.

The initial analysis focused on consecutive unisensory stimuli in the random trial sequence. Within a 2 × 2 framework, there are four possible sequences (Fig. 3a): no switch (same animal and modality), animal switch (same modality, different animal), modality switch (same animal, different modality), and complete switch (different animal and modality). Using mean response times, we performed a 2 × 2 repeated measures ANOVA with animal sequence (repeat, switch) and modality sequence (repeat, switch) as factors (Fig. 3b; an average of 32 trials per participant contributed to each analysis cell). We found a main effect of modality sequence (F_(1,20)_ = 71.26, *p* < 0.001, η_p_^2^ = 0.78), a main effect of animal sequence (F_(1,20)_ = 22.87, *p* < 0.001, η_p_^2^ = 0.53), and an interaction between the two factors (F_(1,20)_ = 12.25, *p* = 0.002, η_p_^2^ = 0.38). The interaction effect drove the main effect of animal sequence, as the effect of animal sequence was significant when the modality was repeated (difference: 32.02 ± 4.99 ms, *p* < 0.001) but not when the modality was switched (difference: 3.64 ± 5.99 ms, *p* = 0.550). This analysis showed that unisensory response times are affected by modality switches, which are modulated by switches in the animal category. Notably, unisensory responses following a switch in modality were on average 42.88 (± 5.08) ms slower than those following a repeat (with the effect being larger in audition than in vision). Hence, the trial sequence effect with unisensory stimuli is of similar magnitude to the RSE itself.

**Figure 4:**
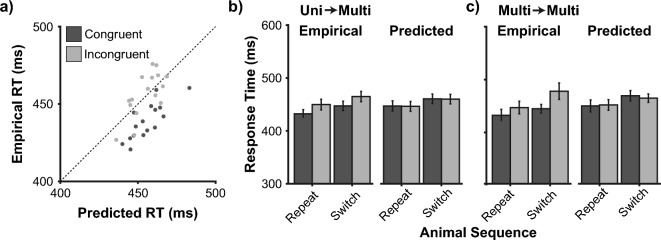
Sequential effects in multisensory response times. (**a**) Empirical response times as a function of independent race model predictions (under consideration of trial sequence). Each data point is the mean response time in one of the four multisensory conditions following one of the eight stimulus conditions (32 data points in total). (**b**) Multisensory response times on trials following unisensory signals. Current multisensory signals were either congruent or incongruent. The animal in the modality presented on the previous unisensory trial was either repeated (e.g., ADVC following AD is an incongruent repeat) or switched (e.g., ADVD following VC is an congruent switch). (**c**) Multisensory response times on trials following multisensory signals. Animals in both modalities are either repeated (e.g., ADVD following ADVD is a congruent repeat) or switched (e.g., ACVD following ADVC is an incongruent switch). Mean (± SEM) of 21 participants.

When computing the predicted RSE using the independent race model, we assumed that unisensory response times are statistically independent. This assumption is clearly violated given the trial sequence effect in unisensory response times. It is therefore possible that the congruency modulation of multisensory response times stems from trial sequence effects and redundancy. To account for this possibility, we investigated trial sequence effects in multisensory responses as a function of congruency and animal switches. In addition to empirical response times, we included in this analysis multisensory response times as predicted from the independent race model but taking the signal presented on the previous trial into account (empirical by predicted multisensory response times are shown in Fig. 4a).

The second analysis of sequence effects looked at current multisensory trials that were preceded by a unisensory trial (Fig. 4b; an average of 17 trials per participant contributed to each empirical analysis cell). As one factor, we considered the congruency of the current multisensory trial. As a second factor, for the modality repeated from the previous unisensory trial, we checked if the animal in this modality was repeated or switched. For example, if a VD precedes an ADVD stimulus, it would be classified as a congruent repeat. A VD preceding an ADVC stimulus would be classified as an incongruent switch. We extended this analysis to include predicted multisensory response times, as discussed in the previous paragraph. Thus, we conducted a 2 × 2 × 2 Repeated Measures ANOVA on response times with congruency (congruent vs incongruent), animal sequence (repeat vs. switch) and dataset (empirical vs. predicted) as factors. We found a significant main effect of animal sequence, multisensory response times with animal repetitions (443.88 ± 8.87 ms) were faster than with animal switches (458.21 ± 8.80 ms) both empirically and predicted (F_(1,20)_ = 32.12, *p* < 0.001, η_p_^2^ = 0.62). A significant main effect of congruency of the current trial was also present (F_(1,20)_ = 30.38, *p* < 0.001, η_p_^2^ = 0.60). However, we found a significant dataset by congruency interaction (F_(1,20)_ = 25.82, *p* < 0.001, η_p_^2^ = 0.56), where the congruency effect was present in the empirical dataset (congruency difference: 17.58 ± 3.29 ms;* p* < 0.001) but not in the predicted response times (congruency difference: 0.65 ± 0.55 ms;* p* = 0.246). No other effects reached significance (all *p*s ≥ 0.240). Therefore, we found an animal sequence effect in multisensory responses that follow unisensory trials, which was predicted by redundancy and the corresponding sequence effect in unisensory responses. In contrast, the congruency effect was not predicted by the independent race model even when considering the signal presented on the previous trial.

For the third analysis, we looked at current multisensory trials preceded by a multisensory trial (Fig. 4c). Here, we considered only a subset of multisensory trials, where both animal stimuli in both modalities are switched or repeated (an average of 8 trials per participant contributed to each empirical analysis cell). Again, we considered the congruency of the current multisensory trial. For example, if an ADVD precedes an ADVD stimulus, it would be classified as a congruent repeat. If an ACVD precedes an ADVC stimulus, it would be classified as an incongruent switch. A 2 × 2 × 2 Repeated Measures ANOVA was conducted on response times with factors congruency (congruent, incongruent), animal sequence (repeat, switch), and dataset (empirical, predicted). Again, we found a significant main effect of animal sequence, multisensory response times with animal repetitions (442.51 ± 9.22 ms) were faster than with animal switches (461.37 ± 8.80 ms) both empirically and predicted (F_(1,20)_ = 11.59, *p* = 0.003, η_p_^2^ = 0.37). Again, we found a significant dataset by congruency interaction (F_(1,20)_ = 5.39, *p* = 0.031, η_p_^2^ = 0.21), where the congruency effect was present in the empirical dataset (congruency difference: 23.81 ± 11.05 ms;* p* = 0.044) but not in the predicted response times (congruency difference: 1.38 ± 5.31 ms;* p* = 0.797). No other effects reached significance (all *p*s ≥ 0.113). Therefore, we also found animal sequence effects in multisensory following multisensory conditions, which are expected by redundancy and the unisensory trial sequence effects. In contrast, the congruency effect was not predicted by the independent race model even when considering the signal presented on the previous trial.

All three analyses suggest that response times in both unisensory and multisensory trials depend on the preceding trial condition. Although the redundancy and unisensory trial sequence effects predict the animal sequence effects present in multisensory responses, they do not predict the congruency modulation of response times.

### Computational modelling analysis

As a final step, we applied a computational modelling approach to analyse the congruency effect in the RSE^23,24^. It should be noted that the above independent race model predictions using probability summation make two critical assumptions. The first, *context invariance*, states that the processing of a signal in one modality is not affected by the presence or absence of a signal in the other modality^3,27–32^. The same assumption is made when computing Miller’s bound. When Miller’s bound is violated (Fig. 2c,d), it is therefore possible to conclude that at least one of the following is wrong: the race model architecture altogether, or the context invariance assumption. The latter option allows for interactive race model architectures (i.e., where there is some crosstalk between unisensory processes). The second, *statistical independence*, states that the occurrence of a response in one modality does not affect the chances of the occurrence of a response in the other modality^27,28,31,38^. For the assumption of statistical independence to hold, unisensory response times should not differ following an animal/modality switch or repeat (but sequence effects occur, Fig. 3). The presence of trial sequence effects allows for a potential correlation to be considered in race model predictions. In summary, differences between empirical and predicted RSEs could be explained by violating one or both assumptions.

**Figure 5:**
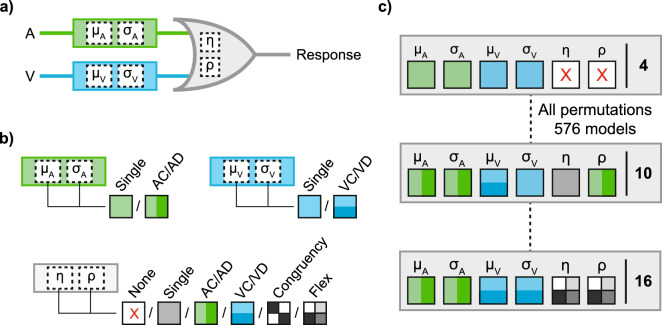
Model design. (**a**) Modified race model. Two parallel unisensory decision units, each including two free parameters (µ and σ) representing the rate and spread of evidence accumulation, are coupled by a logic OR gate. To describe multisensory responses, two interference parameters (ρ and η) account for violations of statistical independence and context invariance. (**b**) Parameter options. Unisensory parameters could vary with animal category, or not. Multisensory parameters could take one of six combinations, ranging from the parameter not being used to the parameter varying for each multisensory condition. (**c**) Candidate models. There are 576 permutations of parameter options. Three examples of candidate models are shown, ranging from only four free parameters (independent race model) to 16 free parameters (the most complex model).

Consequently, we use a modified race model that considers potential violations of context invariance and statistical independence (Fig. 5a). The model describes unisensory response time distributions with two parallel perceptual decision units (each determining if a signal in the corresponding modality is present)^39^. Each unit uses two parameters, the accumulation rate µ and its variability σ, to describe the accumulation of sensory evidence in noise until a threshold is reached and a response is made (a larger accumulation rate leads to faster responses, a larger variability leads to a larger spread of responses). As the critical component of the race model architecture, the two units are coupled by a logic OR gate^20^. Hence, on presentation of a redundant signal, a response on a given trial is triggered by the faster of the two parallel decision units (which leads to the redundancy effect predicted by probability summation). To account for potential violations of the two assumptions described in the previous sections, the modified model includes two further free parameters^23^. The first, the correlation parameter ρ, can account for violations of statistical independence, as revealed by trial sequence effects in unisensory response times (Fig. 3). As a free parameter, the correlation ρ can range between maximal positive (+ 1) and maximal negative (− 1). The second, the additive noise parameter η, allows for violations of context invariance, which enables model predictions to violate Miller’s bound (as found empirically, Fig. 2c,d). As a free parameter, the noise parameter increases the variability in the accumulation of sensory evidence in the unisensory components if a target stimulus is also present in the other modality (i.e., in multisensory compared to unisensory trials). As a technical note, although a noise parameter greater than zero is required for violations of Miller’s bound to occur, the magnitude of violations is modulated by the correlation parameter, with largest violations occurring in case of a maximally negative correlation (− 1). Overall, the modified race model describes response time distributions in the three conditions of the redundant signals paradigm reasonably well^23^.

To analyse the modulation of the RSE by semantic congruency, we extended the above model to the eight stimulus conditions of the 2 × 2 design (Fig. 5b; using an approach recently introduced to study audio-visual motion in depth^24^). Each unisensory parameter (µ_A_, σ_A_, µ_v_, σ_v_) could take either a single value independent of animal category or two separate values to account for response time differences between dog and cat stimuli (e.g., µ_AC_ and µ_AD_ for cat/dog as opposed to only µ_A_ independent of the auditory animal category). For both correlation ρ and noise η parameters, we considered six options to account for response time differences in the multisensory conditions: (1) the parameter is not used and set to 0 (i.e., assuming statistical independence and/or context invariance), (2) a single parameter is used for all four conditions with redundant signals, (3) two parameter values vary with the auditory animal category, (4) two parameter values vary with the visual animal category, (5) two parameter values vary with the congruent/incongruent animal signals, and (6) a different value is used for each of the four conditions with redundant signals (Fig. 5b). To generate a large set of candidate models, we used all 576 permutations of parameter options (2^2^ auditory × 2^2^ visual × 6^2^ multisensory parameters). To account for the response time distributions in all eight conditions, the simplest model had four free parameters, and the most complex had 16 free parameters (Fig. 5c).

**Figure 6:**
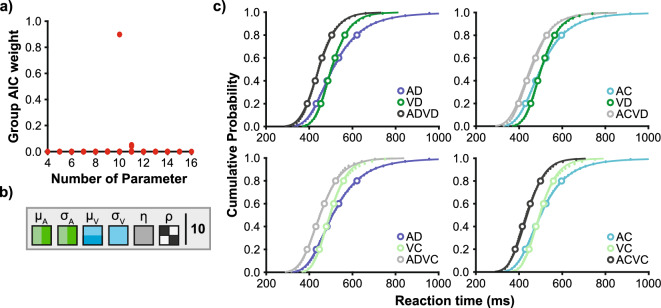
Model fitting and comparison (**a**) Group AIC weights for each of the 576 candidate models as a function of the number of free parameters (weights are close to 0 for most models, i.e. data points are overlapping). The best-fitting model (with the highest weight) has ten free parameters. (**b**) Best-fitting model. Three unisensory parameters vary with unisensory animal stimuli (only σ_V_ does not). The model includes one noise η, the same for all redundant combinations. The model uses two values for the correlation ρ, which varies with semantic congruency—symbols as introduced in Fig. 5. (**c**) Group averaged response time distributions with the best-fitting model (lines). Empirical distributions are shown as response time quantiles (small dots) and quintile estimates used for model fitting (open circles). Each redundant distribution is shown with its unisensory components, which means that every unisensory condition is shown twice.

We fitted all 576 candidate models to the data on the level of individual participants (see Methods). The question is then which model explains the data best, and particularly, which of the multisensory parameters (ρ, η) reflects the modulation of the RSE due to semantic congruency. We used the Akaike information criterion (AIC) to select the best-fitting model, which compares the model fit whilst penalising for more free parameters^40,41^. If the data can be explained to the same level with fewer parameters, then the fewer parameter model was chosen. The best-fitting model included ten free parameters (Fig. 6a; group AIC weight: 0.898; approximately 17.5 times better fitting than the second best-fitting model). The layout of this model included seven unisensory parameters (Fig. 6b; all unisensory parameters except σ_V_ varied with the presented animal category). The model fitted response time distributions in all eight conditions virtually perfectly (Fig. 6c; for best-fitting parameter estimates, see Table 1).

**Table 1:** Best-fitting model parameters. Mean and SEM of 21 participants.

µ_AD_ (s^−1^)	µ_AC_ (s^−1^)	µ_VD_ (s^−1^)	µ_VC_ (s^−1^)	σ_AD_ (s^−1^)	σ_AC_ (s^−1^)	σ_V_ (s^−1^)	ρ_Cong_	ρ_Incong_	η (s^−1^)
1.99 ± 0.05	2.03 ± 0.06	2.01 ± 0.04	2.03 ± 0.04	0.40 ± 0.01	0.39 ± 0.02	0.26 ± 0.01	− 0.27 ± 0.08	0.18 ± 0.08	0.13 ± 0.01

Regarding multisensory interferences, first, the noise parameter η was used in the best-fitting model but did not differ across the four multisensory conditions. A one-sample t-test found that η was significantly larger than zero (t_(20)_ = 12.82, *p* < 0.001), supporting the notion that the context invariance assumption was violated as there was increased variability in the processing of component signals in multisensory conditions. However, the noise parameter was equal across all four multisensory conditions and hence does not explain the semantic congruency effect. Second, the correlation ρ was also used in the best-fitting model and varied with the semantic congruency of the multisensory condition (ρ_C_ and ρ_I_). The presence of the parameter in the model demonstrated a violation of the statistical independence assumption. The correlation for incongruent conditions (0.176) was more positive than for congruent conditions (− 0.269; paired-samples t-test: t_(20)_ = 7.76, *p* < 0.001). Hence, the best-fitting model uses the correlation parameter to describe the congruency modulation of the RSE.

## Discussion

To study the role of semantic content in the RSE, we presented four combinations of audio-visual target animals that were either semantically congruent or incongruent. Critically, audio-visual targets in all four multisensory conditions were redundant in the sense that detection of either auditory or visual animal was sufficient to yield a correct response. All multisensory conditions showed a speed-up of responses compared to the unisensory component signals, which shows that semantic congruency is not required for the RSE to occur (Fig. 2a,b). The absence of multisensory benefits within incongruent conditions in many previous studies^10,12,13,16^ is thus likely due to incongruent signals not being redundant according to the task demands. Yet, the speed-up was increased by about 36% for semantically congruent compared to incongruent combinations, which indicates that semantic content can modulate the RSE.

To rule out that the modulation of the RSE is due to differences in unisensory processing, we used probability summation to quantify the speed-up of responses as expected from the redundancy of targets. To illustrate the issue, for example, a clinical population with deficits in unisensory processing (e.g., slower and more variable responses) may paradoxically show a larger (improved) RSE compared to a control group. However, this paradox could be explained given that probability summation predicts a larger RSE when the variability in unisensory response times is increased (variability rule^5^). As another example, looming biases affect multisensory responses with audio-visual motion-in-depth signals (responses to looming signals are typically faster than responses to receding signals). However, instead of assuming a selective multisensory looming bias, an analysis using probability summation reveals that the modulation of the RSE, in this case, is driven by unisensory looming biases and target redundancy^24^. In the present study, based on the unisensory response times with cat and dog stimuli, probability summation predicted no modulation of the RSE across the four multisensory conditions (Fig. 2a,b). Hence, a factor other than signal redundancy prompts a larger RSE with semantically congruent signals.

As further analysis, we used Miller’s bound^3^ to test if (fastest) response times are still in agreement with race model predictions (under the assumption of context invariance^27–32^). If processes associated with violations of Miller’s bound depended on semantic congruency of targets, as could be derived from the common origin assumption, we had expected to observe violations only in conditions with semantically congruent target combinations. However, we found significant violations of Miller’s bound in all four redundant conditions (Fig. 2c,d). Hence, audio-visual signals do not need to “belong” semantically together to yield an RSE including violations of Miller’s bound. Still, violations were modulated by congruency, with larger violations in congruent compared to incongruent conditions. As a note, despite violations of Miller’s bound at the fast tail of the distribution, the RSE in incongruent conditions overall matched the RSE predicted by the independent race model via probability summation (Fig. 2b). These two findings can be reconciled in that empirical multisensory response times are overall not faster but more variable than predicted by the independent race model. Such increased variability of empirical response times compared to predictions has led to the hypothesis that the context invariance assumption is violated by an unspecific noise interaction between the unisensory decision units^23^. In the modelling approach, we included a corresponding noise parameter, which enables the modified race model to violate Miller’s bound (Fig. 5). In the best-fitting model, this parameter is used but takes the same value for congruent and incongruent targets (Fig. 6). Hence, the model-based analysis provides evidential support for a first main conclusion: The processes associated with violations of Miller’s bound (because the race model architecture, the context invariance assumption, or both are wrong) appear to be separate from the processes leading to the modulation of the RSE by semantic congruency.

When using race model predictions to investigate the RSE, it is important to consider not only the context invariance assumption but also statistical independence, which is assumed in the basic probability summation rule^27,28,31,38^. For statistical independence to hold, signals/performance on a previous trial should not affect responses on a present trial. However, several studies have found sequential effects when the signal modality switches^3,23,33–37^. Here, we found sequential effects involving both the sensory modality (audition, vision) and the animal category (cat, dog) presented on a preceding trial (Fig. 3). Notably, the unisensory sequence effects are of similar magnitude to the RSE itself. Hence, the assumption of statistical independence is clearly violated and must be considered when discussing race model predictions.

As a key question, is it possible that the observed congruency effect is due to multisensory response times inheriting the sequential effects from unisensory responses within the race architecture? Regarding modality switches, this hypothesis seems unlikely because both congruent and incongruent conditions equally contain signals in both modalities. Hence, modality switches and repetitions should affect both conditions equally. However, regarding inheriting animal switches, it is possible that these contribute differentially to multisensory response times in congruent and incongruent conditions. Interestingly, we find that congruency and animal switches affect multisensory reaction times in an additive fashion (Fig. 4). Following Sternberg’s additive factor logic^42^, this suggests that congruency and animal sequence effects are active on independent processing stages. This conclusion is further corroborated by our analysis of predicted response times, which show that the animal sequence effect in multisensory responses is inherited from the corresponding effect in unisensory responses and probability summation (Fig. 4; note that probability summation correctly predicts the reduced magnitude of the animal sequence effect in multi-compared to unisensory responses). In contrast, the congruency effect is not predicted in this way. Hence, this analysis provides evidential support for a second main conclusion: Sequential and congruency effects appear independent, additively affecting response times via different processing stages.

Going back to the modelling approach, as a second free parameter, the correlation ρ has been used to account for modality sequence effects (Fig. 5). In an experiment using semantically unrelated stimuli (random dot motion and arbitrary tones), a strong modality sequence effect was covered by the best-fitting modified race model by assuming a strong negative correlation^23^. The reason for finding a negative correlation is that on a given multisensory trial, which was for example preceded by an auditory stimulus, it is likely to find a relatively fast auditory response compared to a relatively slow visual response (and vice versa for a multisensory trial following a visual stimulus). Hence, the modality sequence effect introduced a negative dependency in the estimated unisensory response time distributions, which need to be considered by race model predictions that are based on these distributions. When using a block design instead, the modality sequence effect necessarily disappeared and the RSE was of smaller magnitude compared to the first experiment with a random trial sequence^23^. The best-fitting model covered this change by assuming a small positive correlation. These results indicated that the correlation parameter was needed and meaningful in accounting for modality sequence effects. Extending this model to the four multisensory conditions here, our best-fitting model uses two values for the correlation parameter to describe the congruency effect. At first sight, this may suggest that the congruency modulation of the RSE is due to trial sequence effects (as in the difference between experiments with random and block designs). However, our previous analysis of animal sequence effects in multisensory responses suggest that sequence and congruency effects are independent and additive. Hence, while the model fits the congruency effect reasonably well with the correlation parameter, it seems that trial sequence effects are not its sole driver, leaving the exact mechanism from a modelling perspective unresolved.

As final items, we speculate about two potential mechanisms that drive the congruency effect. Firstly, based on a similarly designed study, it was previously suggested that participants have slower responses in incongruent compared to congruent trials due to more cautious responding^17^. More specifically, participants responded more accurately but slower during incongruent compared to congruent trials. Using a Drift Diffusion Model^43^, it was established that although evidence for congruent and incongruent stimuli is gathered at the same rate, more evidence was needed to respond in incongruent conditions (higher threshold^17^). Due to the ceiling performance in our experiment, it is not possible to study any differences in error rate between redundant conditions, which implies that our results are rather mute about a potential speed-accuracy trade-off between congruent and incongruent conditions. However, there is debate about whether the decision threshold for responding varies based on the current trial condition, especially when these conditions are presented in random sequences. Commonly, it is believed that a decision threshold is determined before each trial begins^44^ and should not change depending on the unknown trial condition when these conditions are randomised. Consequently, we consider response caution as an unlikely explanation of the congruency effect.

Secondly, processing of incongruent stimuli in a multisensory trial may genuinely interfere with one another. As our modelling approach used two multisensory parameters, this interference needed to be covered by one or the other parameter. Notably, the noise parameter affects particularly the fast tail of the predicted response time distribution (for an illustration of the effect that each model parameter has on predictions, see^23^). Given that the noise parameter in the best-fitting model was the same in congruent and incongruent conditions, it can be concluded that the congruency effect is unlikely to manifest in relatively fast responses. In contrast, the correlation parameter has largest effects on the slow tail of the predicted distribution. Given that the best-fitting correlation parameter was different between congruent and incongruent conditions, it can be concluded that the congruency effect increasingly manifests in relatively slow responses, which is a common finding in Stroop and Flanker tasks^45–47^. Within the parallel race architecture, we speculate that evidence for different animal categories is accumulated in parallel, and that corresponding pools of neurons may exhibit inhibitory connections to pools for different animal categories. On trials where noisy sensory evidence for one target animal builds up relatively fast, there is very little opportunity for the other and more slowly accumulating process to interfere. However, on trials where evidence in both pools builds up at a comparable but rather slow rate, inhibition can mutually delay the time needed for one or the other process to reach its threshold. Such a mechanism would result in a larger congruency effect particularly at the slow tail of the distribution as one stimulus increasingly interferes over time with another's processing in incongruent conditions. Future modelling work may try to explicitly model the effect of such inhibitory connections.

In summary, contrary to previous studies^10,12,13,16^, we have shown that the RSE occurs in conditions with semantically incongruent stimuli. As an important difference, incongruent stimuli in our experiment still contained two redundant targets, which was not the case in the previous studies. Hence, target redundancy is a critical factor for the speed-up of multi- compared to unisensory responses to occur. Yet, the RSE was modulated by semantic content, with semantically congruent conditions showing a larger RSE than incongruent conditions. Overall, our analysis of the RSE at the distribution level and our large-scale modelling analysis have shed new light on the effects of semantic congruency on multisensory decisions. The effect cannot be explained by target redundancy, even when considering trial sequence effects. The effect seems also independent from the processes leading to violations of Miller’s bound, a finding that has dominated research on the RSE over the last decades. We thus conclude that a genuine congruency effect is found, which we speculate is due to an interference mechanism between animal categories.

## Methods

### Participants

A total of 21 participants were recruited. Participants ranged from 18 to 29 years of age (mean: 21.2 years), and all reported normal or corrected-to-normal hearing and eyesight. Participants were compensated with £10 for 2 h, and informed consent was obtained before commencing the experiment. Ethical approval was granted by the *University Teaching and Research Ethics Committee* (UTREC, University of St Andrews; approval code: PS15765). All methods were performed in accordance with the relevant guidelines and regulations.

### Apparatus

The experiment was controlled with an HP Z240 desktop workstation running Windows 10 (Microsoft Corporation) and MATLAB (R2016b) equipped with the Psychtoolbox-3 extension^48–50^. Visual stimuli were presented on a 1920 × 1080 pixels IPS LCD monitor (Cambridge Research System Display++). The monitor refresh rate was 60 Hz. Auditory stimuli were presented through Sennheiser HD-280 Pro over-ear headphones. The auditory sampling rate was 48 kHz. Auditory volume levels were measured using a 2250 Light Brüel and Kjœr sound level meter and an artificial ear (Type 4153). Participants responded using a custom-made, hand-held clicker linked to an RTbox (v5/6^51^). Using the RTbox, onsets of auditory and visual signals were calibrated to be synchronous.

### Experimental design

We utilised the redundant signal paradigm where stimuli were presented in one modality (vision V, audition A) or together (multisensory AV). Participants responded via the clicker on presentation of target animals: dogs (D) and cats (C). Unisensory targets consisted of two auditory (AD, AC) and two visual stimuli (VD, VC). Multisensory targets consisted of two congruent (ADVD, ACVC) and two incongruent conditions (ADVC, ACVD). All multisensory target conditions were redundant (i.e., stimuli in both modalities were targets), so detecting either target was sufficient for a correct response. Participants were asked to withhold a response to other animals (sheep, cow, bird, chicken, frog, and monkey). There were three non-target conditions (auditory, visual, and multisensory catch trials). Unisensory catch trials randomly presented one of the other animals. Multisensory catch trials presented random audio-visual combinations of the catch animals.

### Stimuli

Visual stimuli were eight black-and-white line drawings of animals ^12,25^. All images were constructed from jpegs (96 dpi with equalised luminance) with the animal midpoint at the centre of a 250 × 250 pixels white square. The square was presented at the centre of a grey background screen. Animals alternated between left and right-facing for each visual stimulus presentation to counteract retinal image repetition. Visual stimulus onset was ramped up by increasing the image opacity of every frame by 25% until it was fully opaque at the 4th monitor refresh (i.e., after 50 ms). Moreover, the animal stimuli were presented within random pixel noise added to the stimulus square. For every monitor refresh, 75% of the 250 × 250 pixels had randomly distributed luminance values (ranging from black to white). The dynamic visual background noise was presented throughout each trial.

Auditory stimuli were eight animal sounds (mono WAV-files between 45 and 70kb with bitrates of 1411 kbps, sample rates set to 32 bits and 48 kHz ^12,52^). Auditory stimuli started from the peak of the first wavelength, and in-situ headphone volume was maintained at approximately 65 dB SPL (min = 57.5 dB SPL, max = 69 dB SPL). Auditory signals were embedded in background noise, generated from Gaussian noise filtered using a 1st-order Butterworth band-pass filter with cut-off frequencies of 0.1 and 5 kHz. Auditory background noise was played at 50 dB SPL throughout each trial.

### Procedure

The experimental sessions began by individually presenting the auditory and visual stimuli to the participant to ensure animal name agreement between the two modalities. Participants were then instructed to press the clicker whenever they saw or heard a cat or dog as fast and accurately as possible. To familiarise participants with the task, we presented at least one practice block of 12 trials (all eight possible target trials and four catch trials). Participants had to detect all targets correctly and commit no more than one false alarm to move to the experimental blocks. Otherwise, the practice block was repeated. All participants reached this high-performance level with one practice block.

For each stimulus presentation, a trial began with a minimum foreperiod of 2250 ms in addition to a random component sampled from an exponential distribution with a mean of 250 ms (to counter expectancy). Audio-visual background noise was presented during the entire trial, starting with the foreperiod. After the foreperiod, an animal stimulus was added within the noise. Following a response or after 1500 ms, feedback was provided as a coloured border around the stimulus square (green = hit or correct rejection, red = miss or false alarm). During the feedback, visual noise became static, and the volume of the auditory noise was lowered by 75%. Feedback was presented for 250 ms before the subsequent trial started.

Each participant completed 17 blocks of 74 trials. The first two trials in each block were simply reminders of targets (presented as ADVD and ACVC) and were removed from the analysis. The remaining 72 trials consisted of a random sequence of 48 target trials (six repetitions of the eight target conditions) and 24 catch trials (six repeats of the three catch conditions with randomly selected animals). Across blocks, we presented 102 trials per target condition and participant. Each block lasted about 5 min, and the entire session lasted approximately 1 h and 45 min. Participants were allowed to take breaks between blocks as needed to counter tiredness and were explicitly asked to do so at least after five blocks. With 21 participants, we recorded 25,704 trials, of which 17,136 presented targets.

### Data analysis

The main analyses focused on valid responses to target animals. All incorrect responses during the random trial foreperiod were removed (Table 2; Foreperiod FA). To secure ceiling performance, we had planned to remove participants with a hit rate below 90% from the analysis. However, no participant was removed as all participants achieved a hit rate higher than 97% (mean = 99.85%). We conducted an outlier correction to counter the effect of lapses of attention and/or false alarms that may co-occur close to stimulus onset. Correct response times were transformed to rates (1/RT) to account for the skewed distribution of response times^39^. Then, separately for each participant and condition, response rates that deviated by more than ± 3 × 1.4826 × median absolute deviations from the median were removed as outliers (corresponding to ± 3 standard deviations around the mean with a normal distribution^53^; see Table 2, Outlier; corresponding tools are available as part of the RSE-box^26^). 16,950 target trials from 21 participants remained in the cleaned dataset (100.9 ± 0.1 trials per participant and condition).

**Table 2:** Performance summary. Mean % performance and RTs (± SEM) across 21 participants.

Sensory condition	Trials (#)	Foreperiod FA (%)	FA (%)	Hit (%)	Outlier (%)	Valid RTs (#)	Median RTs (ms)
AD	102	0.00 ± 0.00	–	99.67 ± 0.12	0.61 ± 0.24	101.05 ± 0.24	506 ± 13
AC	102	0.09 ± 0.06	–	99.77 ± 0.15	0.80 ± 0.23	100.86 ± 0.26	500 ± 14
VD	102	0.14 ± 0.08	–	99.91 ± 0.06	1.50 ± 0.34	100.24 ± 0.36	501 ± 10
VC	102	0.00 ± 0.00	–	99.67 ± 0.17	1.36 ± 0.31	100.14 ± 0.39	496 ± 08
AD + VD	102	0.00 ± 0.00	–	99.95 ± 0.05	0.84 ± 0.23	101.10 ± 0.26	443 ± 08
AC + VC	102	0.00 ± 0.00	–	99.95 ± 0.05	0.56 ± 0.13	101.38 ± 0.15	434 ± 08
AC + VD	102	0.00 ± 0.00	–	99.95 ± 0.05	0.47 ± 0.20	101.48 ± 0.20	456 ± 10
AD + VC	102	0.05 ± 0.05	–	99.95 ± 0.05	0.98 ± 0.26	100.90 ± 0.28	449 ± 08
Catch	408	0.02 ± 0.02	3.13 ± 0.40	–	–	–	–

*A* audio, *V* visual, *D* dog, *C* cat, *#* Number of trials, *FA* false alarm.

We performed the analysis of response times on the level of cumulative distribution functions (as shown in Fig. 2a). As a first step, we extracted 50 quantiles for each participant and condition. Based on these distributions, we computed the empirical RSE, the predicted RSE based on probability summation, and violations of Miller’s bound. Corresponding tools are available as MATLAB functions and described in detail as part of the RSE-box^26^.

To analyse trial sequence effects in multisensory response times, we simulated responses using probability summation under consideration of the previous trial (i.e., separately for each multisensory condition and as a function of the previously presented condition). For example, we simulated a response to an ADVD stimulus following an AC trial by randomly sampling from the unisensory constituent signals AD and VD when also following an AC trial. To generate predictions, we sampled with replacement 1000 response times pairs from the two constituents and selected the faster of each pair as the simulated multisensory responses (this method is analogous to computing prediction based on the probability summation rule but seemed more appropriate here given the small trial numbers). Using this approach, we generated 32 separate predictions (four multisensory conditions, each following one of eight experimental conditions). For the trial sequence analysis, we summarized condition depending on the congruency (current signal congruent/incongruent) and animal sequence (repeat/switch).

Repeated measures analysis of variance (ANOVA) was used on reaction times, the RSE, violations of Miller’s bound, and switch cost measurements (see Results for factors). Any significant interactions were investigated using Bonferroni-corrected pairwise comparisons. Two-tailed one-sample and paired-sample t-tests were used to test whether model parameters differed from zero or each other, respectively. The alpha level was set at 0.05.

### Model fitting and comparison

To identify audio-visual interference effects, we applied a computational modelling approach (Fig. 5). At the core, we used a race model architecture, where the faster of two parallel unisensory decision units elicits a response^20,23^. The same approach was recently applied to study audio-visual motion-in-depth^24^. To model responses to unisensory targets, we used the LATER model (linear approach to threshold with ergodic rate^39,54^), where the reciprocal of the response times are normally distributed (recinormal distribution) with parameters µ (mean) and σ (standard deviation). For redundant targets, the model assumes that a response is triggered by the unisensory decision unit, which detects a target first (i.e., the decision unit with the higher rate). Therefore, the exact distribution of responses in the redundant condition is given by the maximum distribution of two Gaussian random numbers^55^. To explain responses to redundant targets, the model includes two multisensory interference parameters^23^. First, instead of assuming statistical independence, the correlation parameter ρ was used to account for trial sequential effects where a response on a given trial depends on the signal/response from a previous trial (e.g., modality switching^23^). Second, instead of assuming context invariance (i.e., presentation of a signal in one modality does not affect processing in the other), the noise parameter η accounts for an unspecific interference between the two constituent decision units (by increasing variability of rates in redundant compared to unisensory trials). This race model has six free parameters to explain response time distributions in the three conditions of the redundant signal paradigm (audition, vision, and redundant). The model is available as part of the RSE-box^26^.

To model the eight target conditions (two auditory, two visual, two congruent, and two incongruent), we fitted a large set of nested models where the six basic parameters could differ with condition (Fig. 5b). For unisensory responses, the LATER parameters (µ, σ) could vary with the animal target type by using two different parameters (e.g., µ_AD_, µ_AC_) or not (e.g., µ_A_). For redundant responses, each of the two interference parameters could take one of six settings: (1) not used, (2) one value for all redundant conditions, (3) two values varying with the auditory animal (AD and AC), (4) two values varying with the visual animal (VD and VC), (5) two values varying with animal congruency (congruent and incongruent), and (6) a different value for each of the four redundant conditions. Using all permutations of parameter options, we created 576 candidate models (Fig. 5c). Candidate models ranged from probability summation with only four parameters (µ_A,_ µ_V,_ σ_A,_ σ_V_) to the most complex race model with 16 free parameters (μ_AD_, μ_AC_, μ_VD_, μ_VC_, σ_AD_, σ_AC_, σ_VD_, σ_VC_, ρ_ADVD_, ρ_ACVC_, ρ_ADVC_, ρ_ACVD_, η_ADVD_, η_ACVC_, η_ADVC_, η_ACVD_).

We used quantile maximum probability estimation to fit all candidate models to the data (QMPE^56,57^). For this, we computed quintiles of the response time distributions and counts of response times falling in the corresponding bins (see open circles in Fig. 6c). We then searched for parameter values that maximise the quantile probability. This was done by minimising the model deviance given by twice the negative log-likelihood summed across the eight target conditions (using Matlab’s fmincon function). Fitting was performed using multiple sets of start values to avoid local minima in the best-fitting estimates. For µ parameters, we used the best-fitting estimate obtained separately in corresponding unisensory conditions and values falling ± 2% apart. For σ parameters, start values used the best-fitting estimate as well as values falling ± 2.5% and ± 5% apart. For ρ parameters, we used ten start values evenly spaced, ranging between − 0.9 and 0.9. For η parameters, we used four start values evenly spaced between 0 and 30% of the best-fitting σ estimates in unisensory conditions. Therefore, fitting of each model was initiated with up to 600 sets of start values (fewer sets were used for models without ρ and/or η parameters). Model fitting was performed separately for each participant. To select the “best” model, we calculated the Akaike Information Criterion (AIC^41^), which balances model fit with the number of free parameters (i.e., model complexity). To select the overall best-fitting model, we computed for each candidate model the group AIC by summing AIC values across participants^58^. Furthermore, group AIC weights were calculated for all 576 candidate models, and the model with the highest weight was selected as the best-fitting^40,59^.

## Data Availability

The research data underpinning this publication can be accessed at 10.17630/a8244449-b097-4080-9871-b26db061de94^60^.
